# Engineering of episomal plasmid structure to enhance non-viral Poly(beta-amino ester) nanoparticle gene delivery to liver and brain cancer cells

**DOI:** 10.1371/journal.pone.0352468

**Published:** 2026-07-23

**Authors:** Joanna Yang, Jack Kollings, Ethan Idnani, David R. Wilson, Isabella G. Cozzone, Shanelle Mendes, Mahita Varanasi, Stephany Y. Tzeng, Jordan J. Green

**Affiliations:** 1 Department of Biomedical Engineering, Johns Hopkins University School of Medicine, Baltimore, Maryland, United States of America; 2 Translational ImmunoEngineering Center, Translational Therapeutics and Regenerative Engineering Center, Johns Hopkins University School of Medicine, Baltimore, Maryland, United States of America; 3 Department of Oncology and the Sidney Kimmel Comprehensive Cancer Center, Johns Hopkins University School of Medicine, Baltimore, Maryland, United States of America; 4 Department of Chemical & Biomolecular Engineering, Johns Hopkins University, Baltimore, Maryland, United States of America; 5 Department of Ophthalmology, Johns Hopkins University School of Medicine, Baltimore, Maryland, United States of America; 6 Department of Neurosurgery, Johns Hopkins University School of Medicine, Baltimore, Maryland, United States of America; 7 Department of Materials Science and Engineering, Johns Hopkins University, Baltimore, Maryland, United States of America; 8 Institute for NanoBioTechnology and the Bloomberg~Kimmel Institute for Cancer Immunotherapy, Johns Hopkins University, Baltimore, Maryland, United States of America; Fudan University, CHINA

## Abstract

The objective of this study was to create and evaluate episomal plasmids for use in non-viral polymeric gene delivery to cancer cells. A comparative analysis was conducted utilizing poly(beta-amino ester)s (PBAEs) as the nanocarrier vector and various hepatocellular carcinoma (HCC) and brain cancer cell lines. A total of fourteen reporter plasmids that varied in promoter (CMV, CAG, EF1α), backbone (Z1, pUNO1, Nanoplasmid [NanoP]), length (~2000 to ~7000 base-pairs), and antibiotic selection marker (Kanamycin, Zeocin, Blasticidin, or none) were constructed, characterized, and evaluated for gene delivery performance. GFP and mCherry plasmids were evaluated in six HCC lines: human (Hep3B), murine (Hepa1–6, Hepa1c1c7), and porcine (A92, A272, B239). Luciferase plasmid constructs were evaluated in three brain cancer lines: murine glioma (CT-2A), human astrocytoma (CCF-STTG1), and human meningioma (IOMM-Lee). When comparing promoters within the same backbone (Z1), the GFP nanoparticles (NPs) under control of a CMV promoter demonstrated consistently higher expression than those with a CAG or EF1α promoter. As EF1α promoters are often used for *in vivo* applications due to their resistance to gene silencing, four GFP plasmids under control of the EF1α promoter were also compared: Z1-EF1α-GFP, pUNO1-GFP, pUNO1-GFP-SV40, and NanoP-GFP. Of these, the NPs delivering NanoP-GFP, a minimal plasmid, resulted in the highest %GFP+ cells in five HCC cell lines, the highest GFP geometric mean fluorescence intensity (gMFI) in all six HCC cell lines, and the highest luciferase signal in two out of three brain cancer cell lines. NanoP was also the only plasmid evaluated without an antibiotic resistance gene, which may be advantageous when selecting a plasmid best able to meet regulatory guidance for clinical translation. Finally, a modest negative correlation between plasmid size and either transfection efficacy or GFP gMFI was observed. Overall, this work helps to inform episomal plasmid design strategy for use with non-viral gene therapies.

## Introduction

Non-viral gene delivery is a rapidly evolving, modular platform for cellular engineering and translatable therapeutics [1–4]. However, non-viral systems face intracellular barriers, including serum instability, endosomal escape, cytosolic transport, and nuclear entry, which together limit transgene levels and reproducibility across cell lines [5–9]. Additionally, expression efficacy often varies with vehicle design, promoter choice, and cellular context. Progress calls for systematic, head-to-head comparisons that separate the contributions of the delivery material from those of the expression plasmid.

Two cancer settings underscore this need. Hepatocellular carcinoma (HCC) is a leading cause of cancer death, with marked molecular heterogeneity and therapeutic constraints imposed by underlying liver disease [10–13]. At the delivery interface, liver-resident macrophages (Kupffer cells) and the sinusoidal endothelium sequester and filter nanoparticles, often reducing their exposure to tumors [6,14]. Delivery of therapeutics to brain tumors is classically limited by the blood–brain barrier, and lineage-specific tumors have variable resistance to non-viral transfection [5–9]. Because of the many intracellular delivery steps involved in gene delivery, including DNA protection [15], cellular uptake [16], endosomal escape [17], transport through the cytosol to the nucleus [18], nuclear import [19,20], transcription, and translation, engineering an optimized nanoparticle requires parallel optimization of both the vehicle chemistry and the plasmid architecture to reach the right cells and sustain the desired transcriptional output in those cells.

Within non-viral platforms, poly(β-amino ester) (PBAE) nanoparticles stand out as promising for plasmid DNA delivery [4,21,22]. PBAEs are synthetically tunable and biodegradable cationic polymers that assemble with DNA through electrostatic interactions [14,23–26]. Their ester bonds enable rapid hydrolysis, which can limit intracellular persistence and reduce cytotoxicity, and their high density of protonatable amines support endosomal buffering that promotes cytosolic access [23,25]. Combinatorial libraries have mapped structure–activity relationships linking monomer choice and end-group chemistry to uptake and expression across diverse cell types [25]. These efforts, complemented by focused optimization studies, show that PBAEs can match or exceed the performance of conventional polycations and lipid-based NPs, such as polyethyleneimine [24,27] and Lipofectamine [4,27], while offering improved tolerability [14,23]. PBAEs have supported high-yield recombinant protein production in mammalian cells and enabled functional intracellular delivery for genome editing [14,24]. In the context of HCC and brain tumors, PBAEs have also been optimized to overcome delivery barriers and concentrate plasmid payloads while limiting off-target transfection [6,26].

In addition to optimizing the biomaterial that forms a gene delivery nanocarrier, the encapsulated expression plasmid’s design is also critical. Work in mammalian systems shows that promoter strength and epigenetic susceptibility, introns, untranslated regions, and polyadenylation signals all influence nuclear import, transcription, and mRNA stability [1–3]. Promoter hierarchies can change across cell types, which means construct performance is context-dependent and must be verified in the intended model [3]. Beyond preclinical studies, plasmid engineering is a standard tool in modern gene therapy: tissue-biased promoters, codon optimization, and regulatory elements that modulate expression have been used to raise efficacy and reduce off-target activity [5,8,9]. These principles motivate the evaluation of plasmid design when adapting non-viral delivery to new tumor lineages.

Building on this rationale, the current work demonstrates the effect of various plasmid designs across panels of liver and brain cancer cell lines. To achieve this, linear PBAEs were fabricated to encapsulate plasmids with a variety of designs and that expressed a fluorescent protein or luciferase reporter. By drawing from both hepatocellular and neural-lineage cell samples, this study evaluates non-viral gene delivery performance across a variety of cells. The study investigates whether the gene expression is primarily limited by the plasmid or by the carrier and reveals trends that can guide plasmid construct design choices. The newly-made plasmids have been deposited at Addgene for accessibility and are described here as a practical reference for laboratories studying non-viral plasmid delivery, particularly in HCC and brain tumor models. This same design logic—choosing an expression plasmid suited to the target cell and pairing it with a vehicle tuned for access—can also inform non-viral strategies in other solid tumors, organoid and engineered tissue systems, and immunoengineering applications.

## Materials and methods

### Plasmid construct cloning

The pUNO1 constructs were generated using the pUNO1 backbone (Invivogen, catalog no. pUNO1-mcs), and clonal genes for GFP, luciferase, and mCherry were purchased from Twist Biosciences (**S1 Table**). The pUNO1 backbone and clonal genes were double digested with NheI-HF (NEB [New England Biolabs], catalog no. R3131) and BamHI-HF (NEB, catalog no. R3136). Ligation was performed with T4 DNA Ligase (NEB, catalog no. M0202S), and plasmid sequences were verified via Sanger sequencing.

Z1 plasmid constructs were generated as previously published [28]. The CMV promoter and EF1α promoter sequences were obtained from the pUNO1-m41BBL sequence (Invivogen, puno1-mtnfsf9) and cloned into the Z1 plasmid. pEGFP-N1 was purchased from Clontech (catalog no. 6085−1), and pcDNA3-fLuc was a gift from William Kaelin (Addgene plasmid #18964).

NanoP-GFP and NanoP-fLuc were purchased from Aldeveron with the EF1α promoter sequence.

Plasmids are also available on Addgene (**Table 1**).

**Table 1:** Addgene plasmid ID.

ID	Plasmid name
135612	Z1-EF1a-GFP
135049	Z1-CMV-GFP
135046	Z1-CAG-GFP
252593	pUNO1-GFP
252592	pUNO1-SV40-GFP
135048	Z1-CMV-AausFP1
135047	Z1-CAG-AausFP1
252596	pUNO1-fLuc
252597	pUNO1 mCherry

### Cell lines

All media for cell line culturing was made with 10% fetal bovine serum (FBS) (MilliporeSigma, catalog no. F4135-500ML) and 1% penicillin/streptomycin (Thermo Fisher Scientific, catalog no. 15140122), and all cells were kept at 37°C and 5% CO_2_.

Hep3b cells were purchased from ATCC (catalog no. HB-8064) and cultured in Eagle’s Minimum Essential Medium (EMEM) (Quality Biological, catalog no. 112-018-101). Hepa1c2c7 cells were purchased from ATCC (catalog no. CRL-2026) and cultured in Alpha minimum essential medium without nucleosides (MEMα) (Thermo Fisher Scientific, catalog no. 12561–056). Hepa1–6 cells were purchased from ATCC (catalog no. CRL-1830). The pig HCC cell lines A92, A272, and B239 cells were purchased from Sus Clinicals (Chicago, IL). The Hepa1–6, A92, A272, and B239 cells were cultured in Dulbecco's Modified Eagle's Medium (DMEM) (Thermo Fisher Scientific, catalog no. 11965092).

CT-2A cells were generously provided by the Betty Tyler lab (Johns Hopkins University) and cultured in DMEM. IOMM-Lee (catalog no. CRL-3370) and CCF-STTG1 (catalog no. CRL-1718) cells were purchased from ATCC and cultured in RPMI 1640 (Thermo Fisher Scientific, catalog no. 11875093).

### Polymer synthesis

One backbone monomer (B4 or B5) was mixed with one sidechain monomer (S3 or S5) and reacted without additional solvent at 85°C overnight at a 1.1:1 backbone-to-sidechain molar ratio. Subsequently, a molar excess of one endcap monomer (E6 or E39) was reacted with the acrylate-terminated polymer in anhydrous tetrahydrofuran (THF) at 25°C for 2 hr. Next, the polymer was then precipitated in anhydrous diethyl ether, washed twice, and stored in anhydrous dimethyl sulfoxide (DMSO) with desiccant at −20°C. Monomers are listed in **Table 2**.

**Table 2:** PBAE monomer components.

Polymer component	Monomer	Chemical Name	Manufacturer	CAS No.
**5-3-6**				
Backbone	B5	1,4-pentanediol diacrylate	Monomer-Polymer and Dajac Labs	36840-85-4
Sidechain	S3	3-amino-1-propanol	Alfa Aesar	156-87-6
Endcap	E6	2-(3-aminopropylamino)ethanol	Sigma Aldrich	4461-39-6
**4-5-39**				
Backbone	B4	1,4-butanediol diacrylate	Alfa Aesar	1070-70-8
Sidechain	S5	5-amino-1-pentanol	Alfa Aesar	2508-29-4
Endcap	E39	1-(2-aminoethyl)piperazine	Alfa Aesar	140-31-8

### Nanoparticle formulation and transfection

On day 0, 10,000 cells per well were seeded into two plates, one for the viability assay, and one for the transfection delivery efficacy assay (96- well, flat bottom plate). On day 1, PBAE 5-3-6 was used to transfect HCC cell lines, and PBAE 4-5-39 was used to transfect the brain cancer cell lines with 600 ng of DNA (pUNO1-GFP) per well in 20 µL of nanoparticles. 20 µL was selected based on prior published protocols for PBAE NP delivery [4,27,28]. In Hep3b cells, nanoparticles were formulated at 30, 60, 90, 120 w/w (weight/weight PBAE polymer-to-DNA mass ratio). In IOMM-Lee cells, nanoparticles were formulated with 600 ng of DNA (pEGFP-N1) at 30, 60, and 90 w/w. These were complexed in 25 mM sodium acetate buffer (pH 5) for 10 minutes. Two hours after transfection, all media was replaced with fresh media. On day 2, the (3-(4,5-dimethylthiazol-2-yl)-5-(3-carboxymethoxyphenyl)-2-(4-sulfophenyl)-2H-tetrazolium) (MTS) assay was conducted using The CellTiter 96® AQueous One Solution Cell Proliferation Assay kit (Promega, catalog no. G3582) following the manufacturer’s protocol on one of the plates, and viability is reported as metabolic activity normalized to untreated cells. On day 3, cells from the second plate were harvested for flow cytometry (Thermo Fisher Scientific Attune NxT) or luciferase assay readouts (Thermo Fisher Scientific, catalog no. 16197). For flow cytometry, cells were washed with PBS, trypsinized with 50 μL, quenched with 150 μL FACS buffer (PBS + 2% FBS) and transferred into a 96-well V-bottom plate (Corning, catalog no. 3897) before spinning down and resuspending in 100 μL FACS buffer. Luciferase assays were conducted according to the manufacturer’s protocols. Further transfections comparing reporter gene expression were conducted using the 90 w/w nanoparticle formulation in HCC cells and the 60 w/w nanoparticle formulation in brain cancer cells.

Nanoparticles formed using 600 ng of plasmid and PBAE 5-3-6 at 60 w/w in 20 μL were also diluted 10x, and the diameter and zeta potential were measured using a ZetaSizer Pro (Malvern Panalytical) after dilution in 0.1x PBS. While N/P ratio is commonly used in other polymer systems, PBAEs are commonly described by w/w instead of N/P. Unlike earlier polymers such as polylysine, almost all the amines found in PBAE structures are tertiary amines and uncharged at pH 7, so N/P ratio does not correspond directly to the molar ratio of positively charged to negatively charged groups [29]. However, Table 3 has been provided for a comparison of N/P ratio and w/w for each nanoparticle. N/P ratios were calculated by dividing the number of amine molecules by the number of phosphate molecules. Calculating N: Polymer mass delivered was divided by the polymer average molecular weight to find the number of polymers. The number of repeating polymer units was then calculated using molecular weights of the repeat unit, endcaps, and polymer average molecular weight. The number of amine molecules in the repeat unit was multiplied by the degree of polymerization, added to the number of amine molecules in the endcaps, and then multiplied by the number of polymers. Calculating P: DNA mass delivered was divided by 330 g/mol (average nucleotide molecular weight per phosphate group).

**Table 3:** Nanoparticle W/W and N/P ratios PBAE.

	w/w	N/P
5-3-6	30	40
5-3-6	60	80
5-3-6	90	120
5-3-6	120	160
4-5-39	30	50
4-5-39	60	100
4-5-39	90	150

### Statistical analysis

Specific statistical tests performed for each data set are described in each figure caption. Each cell experiment represents four independent biological replicates. Flow cytometry files from the Thermo Fisher Scientific Attune NxT were exported into FlowJo (Version 10) for gating. Statistical analysis and figure generation were conducted in Graphpad Prism 10. Gating strategy entailed separating out debris from cells (FSC/SSC), selecting for single cells (FSC-H/FSC-A), and then gating around a negative cell population (BL1-H/SSC-H for GFP, or YL2-H/SSC-H). For the flow cytometry analysis, geometric mean values were preferred over arithmetic means due to the observed log-scale distribution of fluorescence signals. Additional detailed statistical analysis is available in **S2 Table**.

## Results

**Fig 1:**
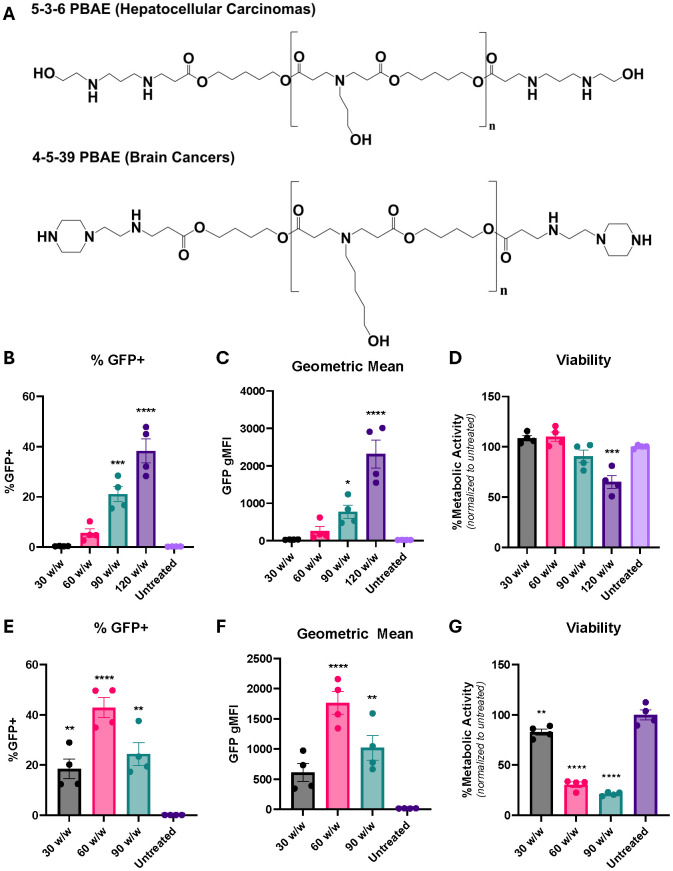
PBAE polymer w/w (weight/weight polymer-to-DNA mass ratios) comparisons in HCC and brain cell lines. **(A)** Polymer structures for 5-3-6 and 4-5-39. **(B)** Percent of cells GFP+ in Hep3b cells after transfection of 5-3-6 across four increasing w/ws (one-way ANOVA, Dunnett’s post test compared to untreated). **(C)** GFP geometric mean fluorescent intensity in Hep3b cells (one-way ANOVA, Dunnett’s post test compared to untreated). **(D)** Viability in Hep3b cells as measured by MTS assay (one-way ANOVA, Dunnett’s post test compared to untreated). **(E)** Percent of IOMM-Lee cells GFP+ after transfection of 4-5-39 across three increasing w/ws (one-way ANOVA, Dunnett’s post test compared to untreated). **(F)** GFP geometric mean fluorescent intensity in IOMM-Lee cells (one-way ANOVA, Dunnett’s post test, compared to untreated). **(G)** Viability in IOMM-Lee cells as measured by MTS assay (one-way ANOVA, Dunnett’s post test compared to untreated). Significance is represented by *P ≤ 0.05, **P ≤ 0.01, ***P ≤ 0.001, and ****P ≤ 0.0001. Data bars shown represent mean ± SEM, n = 4 from one independent experiment.

Human hepatocellular carcinoma (Hep3b) cells were transfected with 5-3-6 PBAE nanoparticles [30], while human meningioma cells (IOMM-Lee) were transfected with 4-5-39 PBAE nanoparticles (**Fig 1A**). Percent transfection by formulation w/w is shown in **Fig 1B**. GFP geometric mean fluorescence intensity (gMFI) and viability are also reported (**Fig 1C-1D**). As w/w increases, transfection efficacy and GFP gMFI increase while viability decreases. In this context, efficacy is defined as expression of the delivered reporter gene. While nanoparticles formed at 120 w/w have the highest transfection efficacy at 38 ± 5% (mean ± SEM), the viability decreases to 65 ± 6%. Although nanoparticles formed at 90 w/w have lower transfection efficacy compared to those formed at 120 w/w, with 21 ± 3% cell transfected, they allow higher viability with 91 ± 6% survival, which is not significantly lower than that of the untreated group. Thus, the 90 w/w nanoparticle was selected for further usage in HCC cells. We did not evaluate plasmid efficacy across all transfection conditions. However, given that 1) nanoparticle formulation primarily governs cellular uptake and intracellular delivery efficacy, 2) plasmid design determines downstream transcriptional and translational output, and 3) plasmid expression occurs downstream of nanoparticle delivery, we expect that relative plasmid rankings would be similar using different nanoparticle formulations. In IOMM-Lee cells, transfection efficacy peaked with nanoparticles formed at 60 w/w, with 43 ± 4% GFP+ cells (**Fig 1E**), and a similar trend was observed in the GFP gMFI (**Fig 1F**), with the expected inverse trend in viability (**Fig 1G**)**.** The 60 w/w condition was selected for further usage in brain cancer cells due to the higher transfection efficacy.

**Fig 2:**
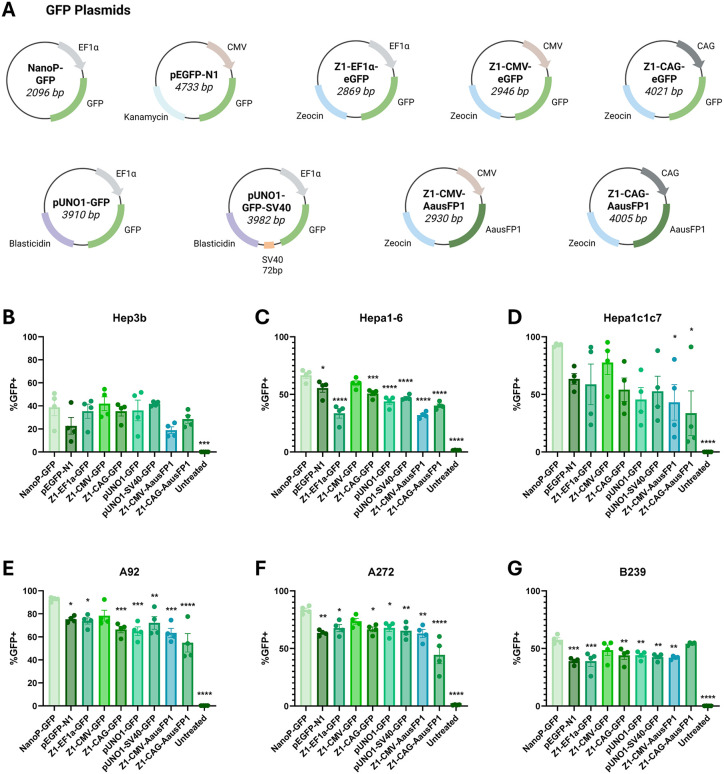
Transfection of various GFP plasmid constructs into six HCC cell lines. **(A)** GFP plasmid construct maps. **(B)** Transfection of GFP plasmids into Hep3b cells, **(C)** Hepa1-6 cells, **(D)** Hepa1c1c7 cells, **(E)** A92 porcine HCC cells, (**F)** A272 porcine HCC cells, and **(G)** B239 porcine HCC cells. All statistics performed are one-way ANOVA, Dunnett’s post test compared to NanoP-GFP. Significance is represented by *P ≤ 0.05, **P ≤ 0.01, ***P ≤ 0.001, and ****P ≤ 0.0001. Data bars shown represent mean ± SEM, n = 4 from one independent experiment.

We tested nine GFP plasmids that vary in promoter (CMV, CAG, EF1α), backbone (Z1, pUNO1, NanoP), size, and antibody selection gene (kanamycin, zeocin, blasticidin, or none) across six HCC lines: human (Hep3B), murine (Hepa1–6, Hepa1c1c7), and porcine (A92, A272, B239) (**Fig 2A**). These cell lines were transfected with the PBAE nanoparticle selected in Fig 1 (600 ng DNA, 90 w/w, in 20 µL), each formulated with a different plasmid construct to compare gene expression. The pEGFP-N1 plasmid is a commercially available construct containing a 795 base-pair kanamycin resistance gene, driven by the 589 base-pair CMV promoter [31]. The Z1 plasmids contained a shorter antibiotic resistance gene, zeocin, 382 base-pairs in length, and pUNO1 plasmids contained blasticidin, 425 base-pairs in length. Nanoparticle size and zeta potentials can be found in S1 Fig in S1 File.

In Hep3b cells, NPs formed with Z1-CMV-GFP transfected 42 ± 6% of cells, and NPs formed with pUNO1-GFP-SV40 transfected 42 ± 1% of cells, making them the highest-expressing plasmids. NPs formed with NanoP-GFP demonstrated slightly lower transfection (39 ± 7%, **Fig 2B**). In the murine HCC cell lines, NPs formed with NanoP-GFP consistently demonstrated the highest transfection, followed by NPs formed with Z1-CMV-GFP. In Hepa1–6 cells that received NanoP-GFP NPs, 66 ± 3% of cells were GFP + , and in those that received Z1-CMV-GFP, 60 ± 2% of cells were GFP+ (**Fig 2C**). In Hepa1c1c7 cells that received NanoP-GFP NPs, 93 ± 1% of cells were GFP + , and in those that received Z1-CMV-GFP, 80 ± 10% of cells were GFP+ (**Fig. 2D**). Similarly, in the three porcine HCC cell lines, NanoP-GFP NPs exhibited the highest transfection, with 93 ± 1% in A92 cells (**Fig 2E**), 83 ± 2% in A272 cells (**Fig 2F**), and 57 ± 2% in B239 cells (**Fig. 2G**). Z1-CMV-GFP NPs exhibited the second highest transfection in A92 and A272 cells, with 78 ± 5% GFP+ in A92 cells and 74 ± 2% GFP+ in A272 cells, although this trend did not extend to B239 cells. In B239 cells, Z1-CAG-AausFP1 NPs demonstrated the second highest transfection. Trends between plasmid length and transfection efficacy are plotted in S2 Fig and S3 Fig in S1 File.

**Fig 3:**
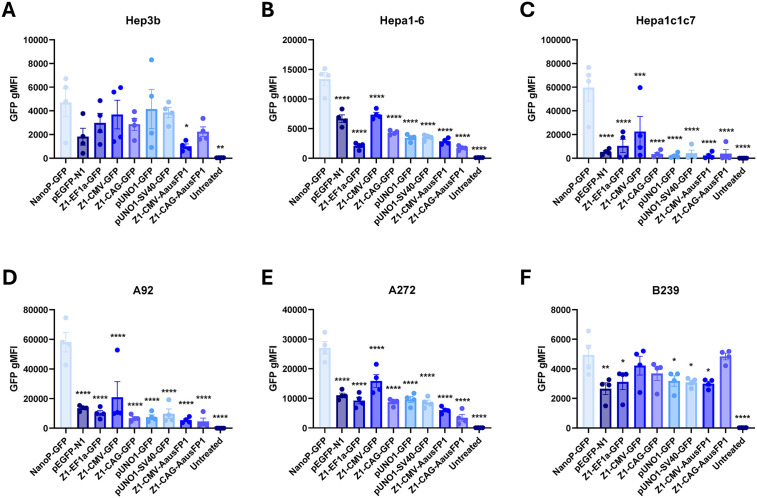
GFP geometric mean fluorescence intensity (gMFI) in HCC cell lines. GFP gMFI in **(A)** Hep3b cells. **(B)** Hepa1-6 cells. **(C)** Hepa1c1c7 cells. **(D)** A92 porcine HCC cells. **(E)** A272 porcine HCC cells, and **(F)** B239 porcine HCC cells. All statistics performed are one-way ANOVA, Dunnett’s post test compared to NanoP-GFP. Significance is represented by *P ≤ 0.05, **P ≤ 0.01, ***P ≤ 0.001, and ****P ≤ 0.0001. Data bars shown represent mean ± SEM, n = 4 from one independent experiment.

While the percent of cells that are GFP + is a common metric to assess gene expression, it remains a binary measure for expression. Mean fluorescence intensity, on the other hand, reflects the level of GFP expression within the cell population. It provides information on how strongly a gene is expressed, rather than simply whether or not a gene is expressed. Together, these metrics give a more holistic understanding of gene expression dynamics in a given cell population. In **Fig 3**, GFP gMFI data is shown for the transfection experiments from **Fig 2**. In Hep3B cells, though NanoP-GFP NPs demonstrated the third highest transfection measured as percent of cells GFP + , they led to the highest GFP gMFI, at 5000 ± 1000 (**Fig 3A**). Z1-CMV-GFP NPs, with the highest percent of cells transfected, led to a GFP gMFI of 4000 ± 1000. In Hepa1–6 cells and Hepa1c1c7 cells, NanoP-GFP NPs achieved both the highest percent of GFP+ cells and also the highest gMFI, with a value of 13000 ± 1000 in Hepa1–6 cells and a value of 60000 ± 11000 in Hepa1c1c7 cells (**Fig 3B-3C**). Z1-CMV-GFP NPs achieved a GFP gMFI of 7000 ± 400 in Hepa1–6 cells and a GFP gMFI of 20000 ± 10000 in Hepa1c1c7 cells. This represents a 2-3X increase in GFP gMFI in cells transfected with NanoP-GFP NPs over cells transfected with Z1-CMV-GFP NPs, despite only a 5–15% increase in the percent of cells transfected. Similarly, NanoP-GFP NPs achieved the highest GFP gMFI levels in all three porcine HCC cell lines (**Fig 3D-3F**), with the GFP gMFI for NanoP NPs being several-fold higher than that of the next highest transfecting NP. Trends between plasmid length and GFP gMFI are plotted in S4 Fig and S5 Fig in S1 File.

An SV40 72 base-pair tandem repeat sequence was incorporated into the pUNO1-GFP plasmid to produce pUNO1-GFP-SV40 [32,33]. SV40-derived sequences have been reported to function as transcriptional enhancer elements and have also been implicated in sequence-dependent plasmid nuclear import. Previous literature has found that sequence can act as a nuclear localization sequence as well as an enhancer element that increases transcriptional activity and transgene expression [34,35]. In Hepa1–6, Hepa1c1c7, and A92 cells, we observed a modest increase in the GFP gMFI in cells transfected with pUNO1-GFP-SV40 NPs over those that received pUNO1-GFP NPs, though this trend did not extend to the Hep3B, A272 or B239 cells. For example, the GFP gMFI of cells transfected with pUNO1-GFP-SV40 NPs in A92 cells was higher, at 10000 ± 3000, as opposed to 7000 ± 3000 in cells transfected with pUNO1-GFP NPs. The reverse trend was observed in A272 cells, where the GFP gMFI of cells transfected with pUNO1-GFP-SV40 NPs was lower, at 9000 ± 2000, as opposed to 10000 ± 2000 with pUNO1-GFP NPs.

**Fig 4:**
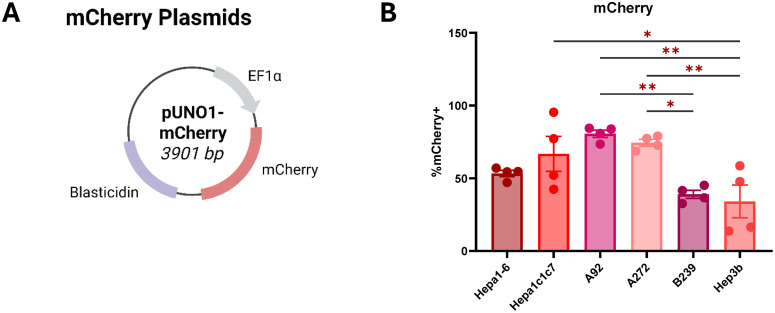
Transfection of pUNO1-mCherry plasmid into six HCC cell lines. **(A)** pUNO1-mCherry plasmid construct map. **(B)** Comparison of mCherry plasmid transfection across HCC cell lines (one-way ANOVA, Tukey's multiple comparisons test). Significance is represented by *P ≤ 0.05, **P ≤ 0.01, ***P ≤ 0.001, and ****P ≤ 0.0001. Data bars shown represent mean ± SEM, n = 4 from one independent experiment.

While GFP is widely used for visualization of protein expression in cells, red fluorescent proteins (RFP) may be preferable for other multicolor applications that require multiple fluorophores. Within RFPs, mCherry is the most widely used due to high brightness and photostability [36]. A mCherry plasmid was cloned into the pUNO1 backbone (**Fig 4A**) and delivered into HCC cell lines to measure differences in expression among cell lines. The pUNO1 backbone was selected for this experiment because it represents a plasmid that can be easily amplified in the lab, without requiring a third-party manufacturer. A92 cells were the most robustly transfected cell line (81 ± 3%), while Hep3B were the least (30 ± 10%) (**Fig 4B**).

**Fig 5:**
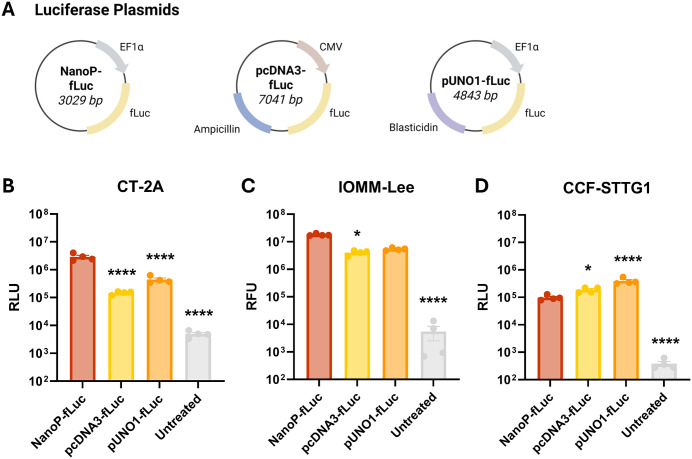
Transfection of various luciferase plasmid constructs into three brain cancer cell lines. **(A)** Luciferase plasmid construct maps. Transfection of luciferase plasmids into **(B)** CT-2A glioma cells, **(C)** IOMM-Lee cells, and **(D)** CCF-STTG1 astrocytoma cells. All statistics performed are one-way ANOVA, Dunnett’s post test comparing all groups to NanoP-fLuc. Significance is represented by *P ≤ 0.05, **P ≤ 0.01, ***P ≤ 0.001, and ****P ≤ 0.0001. Data bars shown represent mean ± SEM, n = 4 from one independent experiment.

While there are many applications for fluorescent genes such as GFP, these proteins require external light for excitation and may be overestimated due to non-specific autofluorescence (e.g., from animal skin). Conversely, bioluminescence reporter genes, such as luciferase, offer higher sensitivity and deeper tissue penetration [37,38]. As such, luciferase is often used as a reporter gene in lieu of fluorescent proteins *in vivo*, such as for *in vivo* imaging [39]. One such application may be for detecting gene expression in brain cancer models or other disease models affecting difficult-to-access organs, where luciferase expression can allow longitudinal monitoring while limiting invasive and complex surgical procedures. Three plasmids encoding luciferase, pcDNA3-fLuc, pUNO1-fLuc, and NanoP-fLuc, were transfected into brain cancer cells using 4-5-39 PBAE NPs at 60 w/w in 20 µL (**Fig 5A**). Three brain cancer cell lines were transfected: murine glioma (CT-2A), human astrocytoma (CCF-STTG1), and human meningioma (IOMM-Lee). Transfection was quantified using a luciferase assay and measured in relative luminescence units (RLUs). NanoP-fLuc had the highest transfection efficacy in the CT-2A cell line at 2.9x10^6^ ± 4x10^5^ RLU (**Fig 5B**) and in IOMM-Lee cells at 17.9x10^6^ ± 8x10^5^ RLU (**Fig 5C**). However, this trend did not extend to CCF-STTG1 cells, where NanoP-fLuc did not outperform all other luciferase constructs, displaying the lowest expression among all three plasmids in CCF-STTG1 cells at 1x10^6^ ± 1x10^5^ RLU (**Fig 5D**).

## Discussion

In this study, we systematically compared a panel of plasmid backbones encoding a fluorescent protein or luciferase. Plasmid designs differed in promoter type, antibiotic resistance genes, and total plasmid size. They were evaluated for transfection efficacy across six HCC cell lines derived from human, mouse, and pig, as well as three brain cancer lines derived from human and mouse. By holding the PBAE delivery platform constant within each cancer model, this study was designed to evaluate how plasmid architecture and cellular context influence reporter gene expression.

Constitutive promoters, such as CMV, CAG, and EF1α, drive gene expression constantly without requiring external signals, although the specific application may influence promoter selection. At less than 600 bp, the CMV promoter is a popular choice for viral vectors that have limitations on genetic cargo size. But although the CMV promoter is the most widely used promoter for recombinant protein production, it has also been reported to be susceptible to gene silencing mechanisms [40–42]. By contrast, the non-viral hybrid EF1α promoter is less prone to silencing than CMV and has gained traction in clinical settings, especially in CAR-T cell therapies [43,44], such as in the case of the FDA approved product Kymriah [45]. This hybrid EF1α promoter is a synthetic promoter combining the short core-EF1α promoter with elements from Human T-Cell Leukemia Virus (HTLV). The CAG promoter is also a hybrid promoter, composed of the CMV enhancer, chicken β-actin promoter, and rabbit β-globin intron, and it is also frequently used in preclinical studies [46]. In our study, we compared these three promoters head-to-head in the Z1 backbone. The plasmids with CMV promoter consistently outperformed those with CAG or EF1α promoters across all six HCC cell lines. The cells receiving Z1-CMV-GFP NPs had a higher percentage of GFP-positive cells and higher GFP gMFI than those treated with Z1-CAG-GFP or Z1-EF1α-GFP. This may reflect favorable matching between CMV regulatory elements and the transcriptional state of the HCC cell lines tested. The CMV major immediate-early enhancer/promoter contains binding sites for transcription factors such as NF-κB, AP-1, and CREB/ATF family members. HCC is frequently associated with inflammatory and stress-responsive transcriptional programs, including NF-κB and AP-1 signaling [47,48]. However, transcription factor binding and promoter occupancy were not directly measured in this study, so this mechanism remains inferential.

The cell-type-specific results also suggest that plasmid performance is influenced by intracellular processing. After PBAE-mediated uptake and endosomal escape, plasmid DNA must traffic through the cytosol and reach the nucleus. Prior work has shown that cellular uptake and nuclear uptake are distinct steps in polymeric gene delivery [49]. Plasmids can also interact with importins, motor-associated proteins, and transcription factors after transfection, which may influence cytosolic transport and nuclear import [50]. In addition, DNA nuclear targeting sequences that bind transcription factors such as NF-κB can enhance non-viral plasmid nuclear import [51]. These studies support the idea that plasmid architecture may affect both intracellular trafficking and transcriptional output.

As the EF1α promoter is less susceptible to gene silencing, plasmids containing this promoter are often chosen for *in vivo* applications [52–54]. In this study, however, expression was measured only at day 3. Therefore, the current data support CMV as the strongest early-expression promoter in the HCC lines tested, but they do not directly compare long-term promoter stability. The potential advantage of EF1α for sustained expression should be interpreted in the context of prior literature rather than inferred from this dataset alone [54,55]. Among the GFP plasmids, four were driven by the EF1α promoter: Z1-EF1α-GFP, pUNO1-GFP, pUNO1-GFP-SV40, and NanoP-GFP. The NanoP plasmid was the shortest plasmid, followed by Z1-EF1α-GFP, pUNO1-GFP, and pUNO1-SV40-GFP. The NanoP plasmid displayed higher transfection efficacy compared to the remaining three plasmids, which may have been due to a higher copy number by mass. Additionally, prior studies have demonstrated that the SV40 72 base-pair sequence promotes nuclear import and consequently transgene expression, especially in non-dividing cells [35,56]. However, between the pUNO1-GFP and pUNO1-GFP-SV40 plasmids, the mean GFP gMFI was only higher in three out of the six HCC cell lines: Hepa1–6, Hepa1c1c7, and A92. Since the SV40-containing construct was not designed to separate enhancer activity from nuclear import activity, these modest increases may reflect transcriptional enhancement, nuclear import effects, or both. Given the rapid division and nuclear envelope breakdown in cancer cells, the impact of the SV40 enhancer on DNA nuclear import may be less pronounced in the context of cancer.

Notably, the NanoP-GFP outperformed other plasmid constructs in most cell lines. In HCC cells, the NanoP-GFP achieved the highest percent of cells GFP+ in five out of six cell lines and achieved the highest GFP gMFI in all six cell lines. Interestingly, the GFP gMFI in cells receiving NanoP-GFP NPs was several-fold higher than other plasmids, even when the %GFP+ cells were similar, reflecting the amount of protein expressed from NanoP-GFP in each cell. Together, %GFP+ cells and gMFI suggest that NanoP’s advantage may not be limited to transfecting more cells. In some cell lines, NanoP produced similar %GFP+ values but higher gMFI, suggesting greater expression per transfected cell. In brain cancer cells, NanoP-fLuc achieved the highest luciferase expression in two of the three cell lines tested. However, NanoP-fLuc did not uniformly outperform the other luciferase constructs across all neural-lineage models, indicating that NanoP performance remains cell-context dependent. In CCF-STTG1 cells, differences in promoter accessibility, nuclear import, episomal persistence, or reporter processing may have limited expression despite the smaller plasmid size. Future mechanistic studies will be needed to define why certain cell types respond differently to minimal plasmid architectures.

The NanoP backbone was the shortest GFP construct tested, at 2096 base-pairs, and also the shortest luciferase construct tested, at 3029 base-pairs. It was also the only plasmid in our study without an antibiotic resistance gene, which offers further advantages in clinical translation, as the FDA regulatory guidance recommends avoiding antibiotic resitance markers where possible due to concerns about horizontal gene transfer and antimicrobial resistance [57–59]. Generally, we observed a negative correlation with increasing plasmid length and transfection efficacy or GFP gMFI, although these trends should be interpreted cautiously. The plasmid library in this study spans approximately 2,000–7,000 bp, and therefore these trends may not extrapolate to larger (>10–15 kbp) constructs. In addition, differences in plasmid backbone size and sequence may alter nanoparticle physicochemical properties, including size and surface charge, which could also influence uptake and expression. Because the plasmids differed in backbone, antibiotic resistance marker, and size, the observed size-performance relationship should be interpreted as an association rather than evidence that plasmid size alone determined expression. Smaller plasmids also delivered a higher number of plasmid molecules per equal DNA mass, which may have contributed to the increased expression observed with NanoP. Finally, because plasmid rankings were not tested across all constructs at 120 w/w, we cannot exclude the possibility that relative performance may shift under higher-dose or more cytotoxic formulation conditions.

These findings provide a practical framework for plasmid selection in non-viral gene therapy. If broad cell coverage is the goal, the percentage of reporter-positive cells should be prioritized. This may be relevant for intracellular proteins, genome-editing enzymes, or tumor reprogramming strategies where the number of modified cells matters. If high per-cell output is needed, gMFI or luminescence may be more informative. This may apply to secreted cytokines, enzymes, or other paracrine therapeutic proteins. Promoter choice should also match the intended expression window. CMV may be useful for strong early expression. EF1α may be preferable when sustained expression or reduced silencing is desired, but this should be tested in the intended model because promoter stability is context dependent. Backbone design should also consider plasmid size, bacterial sequence content, antibiotic resistance markers, and regulatory compatibility. Compact antibiotic-free constructs such as NanoP may be useful starting points, but the present results show that no single plasmid design should be assumed to be optimal across all cell types.

Our comparative analysis of plasmid backbones across various cancer cell lines highlights the impact of promoter choice, plasmid length, antibiotic selection markers, and cellular context on transfection efficacy and gene expression. NanoP is a promising backbone for translational applications due to its high transfection efficacy across many cell lines and compliance with regulatory requirements. Because mCherry was tested only in the pUNO1 backbone, those experiments should be interpreted as cell-line comparisons rather than direct cross-backbone comparisons for the mCherry reporter. While these studies were conducted in established cancer cell lines, the observed transfection trends may not directly translate to primary or patient-derived cells, which have key biological differences such as proliferation rate, nuclear envelope accessibility, and transcriptional activity. Future studies may expand testing to a broader library of plasmids and cell types, as well as evaluating these plasmids in relevant *in vivo* models to better understand transfection efficacy and stability in biological environments. Together, these results support a context-aware approach to non-viral gene therapy design, in which both the delivery material and plasmid architecture are optimized for the intended target cell and therapeutic goal.

## Supporting information

S1 Table Clonal gene sequences ordered from Twist Biosciences. (PDF)

S2 Table Detailed statistical analysis. (XLSX)

S1 File Supporting Information PDF. Contains Figures S1-S5. Figure S1. Biophysical properties of PBAE nanoparticles encapsulating varied DNA plasmids. Figure S2. Correlation between plasmid size and transfection efficacy of six HCC lines. Figure S3. Correlation between plasmid size and transfection efficacy of all cells. Figure S4. Correlation between plasmid size and GFP gMFI of six HCC lines. Figure S5. Correlation between plasmid size and GFP gMFI of all cells.
(PDF)
