# Supplementary material for: Engineering of episomal plasmid structure to enhance non-viral Poly(beta-amino ester) nanoparticle gene delivery to liver and brain cancer cells
Source: PLoS One. 2026 Jul 23;21(7):e0352468. doi: 10.1371/journal.pone.0352468 (PMC13395325; doi:10.1371/journal.pone.0352468)
Supplement: S1 Table — (PDF) [file pone.0352468.s001.pdf]

Table S1: Clonal gene sequences ordered from Twist Biosciences

GFP	gagatcaccggcgtgtcgacggatccatggtgagcaagggcgaggagctgttcaccggggtggtgcccatcctggtcgagct<br>ggacggcgacgtaaacggccacaagttcagcgtgtccggcgagggcgagggcgatgccacctacggcaagctgaccctga<br>agttcatctgcaccaccggcaagctgcccgtgccctggcccaccctcgtgaccaccctgacctacggcgtgcagtgcttcagcc<br>gctaccccgaccacatgaagcagcacgacttcttcaagtccgccatgcccgaaggctacgtccaggagcgcaccatcttcttca<br>aggacgacggcaactacaagacccgcgccgaggtgaagttcgagggcgacaccctggtgaaccgcatcgagctgaagggc<br>atcgacttcaaggaggacggcaacatcctggggcacaagctggagtacaactacaacagccacaacgtctatatcatggccga<br>caagcagaagaacggcatcaaggtgaacttcaagatccgccacaacatcgaggacggcagcgtgcagctcgccgaccacta<br>ccagcagaacacccccatcggcgacggccccgtgctgctgcccgacaaccactacctgagcacccagtccgccctgagcaa<br>agaccccaacgagaagcgcgatcacatggtcctgctggagttcgtgaccgccgccgggatcactctcggcatggacgagctg<br>tacaagtaagctagctggccagacatgataagatac
GFPSV40	gagatcaccggcgtgtcgacggatccatggtgagcaagggcgaggagctgttcaccggggtggtgcccatcctggtcgagct<br>ggacggcgacgtaaacggccacaagttcagcgtgtccggcgagggcgagggcgatgccacctacggcaagctgaccctga<br>agttcatctgcaccaccggcaagctgcccgtgccctggcccaccctcgtgaccaccctgacctacggcgtgcagtgcttcagcc<br>gctaccccgaccacatgaagcagcacgacttcttcaagtccgccatgcccgaaggctacgtccaggagcgcaccatcttcttca<br>aggacgacggcaactacaagacccgcgccgaggtgaagttcgagggcgacaccctggtgaaccgcatcgagctgaagggc<br>atcgacttcaaggaggacggcaacatcctggggcacaagctggagtacaactacaacagccacaacgtctatatcatggccga<br>caagcagaagaacggcatcaaggtgaacttcaagatccgccacaacatcgaggacggcagcgtgcagctcgccgaccacta<br>ccagcagaacacccccatcggcgacggccccgtgctgctgcccgacaaccactacctgagcacccagtccgccctgagcaa<br>agaccccaacgagaagcgcgatcacatggtcctgctggagttcgtgaccgccgccgggatcactctcggcatggacgagctg<br>tacaagtaaggtgtggaaagtccccaggctccccagcaggcagaagtatgcaaagcatgcatctcaattagtcagcaaccagct<br>agctggccagacatgataagatac
Luciferase	gagatcaccggcgtgtcgacggatccatggaagacgccaaaaacataaagaaaggcccggcgccattctatccgctggaaga<br>tggaaccgctggagagcaactgcataaggctatgaagagatacgccctggttcctggaacaattgcttttacagatgcacatatc<br>gaggtggacatcacttacgctgagtacttcgaaatgtccgttcggttggcagaagctatgaaacgatatgggctgaatacaaatc<br>acagaatcgtcgtatgcagtgaaaactctcttcaattctttatgccggtgttgggcgcgttatttatcggagttgcagttgcgcccgc<br>gaacgacatttataatgaacgtgaattgctcaacagtatgggcatttcgcagcctaccgtggtgttcgtttccaaaaaggggttgca<br>aaaaattttgaacgtgcaaaaaaagctcccaatcatccaaaaaattattatcatggattctaaaacggattaccagggatttcagtc<br>gatgtacacgttcgtcacatctcatctacctcccggttttaatgaatacgattttgtgccagagtccttcgatagggacaagacaatt<br>gcactgatcatgaactcctctggatctactggtctgcctaaaggtgtcgctctgcctcatagaactgcctgcgtgagattctcgcat<br>gccagagatcctatttttggcaatcaaatcattccggatactgcgattttaagtgttgttccattccatcacggttttggaatgtttacta<br>cactcggatatttgatatgtggatttcgagtcgtcttaatgtatagatttgaagaagagctgtttctgaggagccttcaggattacaag<br>attcaaagtgcgctgctggtgccaaccctattctccttcttcgccaaaagcactctgattgacaaatacgatttatctaatttacacga<br>aattgcttctggtggcgctcccctctctaaggaagtcggggaagcggttgccaagaggttccatctgccaggtatcaggcaagg<br>atatgggctcactgagactacatcagctattctgattacacccgagggggatgataaaccgggcgcggtcggtaaagttgttcca<br>tttttgaagcgaaggttgtggatctggataccgggaaaacgctgggcgttaatcaaagaggcgaactgtgtgtgagaggtccta<br>tgattatgtccggttatgtaaacaatccggaagcgaccaacgccttgattgacaaggatggatggctacattctggagacatagct<br>tactgggacgaagacgaacacttcttcatcgttgaccgcctgaagtctctgattaagtacaaaggctatcaggtggctcccgctga<br>attggaatccatcttgctccaacaccccaacatcttcgacgcaggtgtcgcaggtcttcccgacgatgacgccggtgaacttccc<br>gccgccgttgttgttttggagcacggaaagacgatgacggaaaaagagatcgtggattacgtcgccagtcaagtaacaaccgc<br>gaaaaagttgcgcggaggagttgtgtttgtggacgaagtaccgaaaggtcttaccggaaaactcgacgcaagaaaaatcagag<br>agatcctcataaaggccaagaagggcggaaagatcgccgtgtaagctagctggccagacatgataagatac

m<br>C<br>he<br>rr<br>y	gagatcaccggcgtgtcgacggatccatggtgagcaagggcgaggaggataacatggccatcatcaaggagttcatgcgcttc<br>aaggtgcacatggagggctccgtgaacggccacgagttcgagatcgagggcgagggcgagggccgcccctacgagggcac<br>ccagaccgccaagctgaaggtgaccaagggtggccccctgcccttcgcctgggacatcctgtcccctcagttcatgtacggctc<br>caaggcctacgtgaagcaccccgccgacatccccgactacttgaagctgtccttccccgagggcttcaagtgggagcgcgtgat<br>gaacttcgaggacggcggcgtggtgaccgtgacccaggactcctccctgcaggacggcgagttcatctacaaggtgaagctgc<br>gcggcaccaacttcccctccgacggccccgtaatgcagaagaagaccatgggctgggaggcctcctccgagcggatgtaccc<br>cgaggacggcgccctgaagggcgagatcaagcagaggctgaagctgaaggacggcggccactacgacgctgaggtcaaga<br>ccacctacaaggccaagaagcccgtgcagctgcccggcgcctacaacgtcaacatcaagttggacatcacctcccacaacga<br>ggactacaccatcgtggaacagtacgaacgcgccgagggccgccactccaccggcggcatggacgagctgtacaagtaggc<br>tagctggccagacatgataagatac
