# Supplementary material for: Engineering of episomal plasmid structure to enhance non-viral Poly(beta-amino ester) nanoparticle gene delivery to liver and brain cancer cells
Source: PLoS One. 2026 Jul 23;21(7):e0352468. doi: 10.1371/journal.pone.0352468 (PMC13395325; doi:10.1371/journal.pone.0352468)
Supplement: S1 File — Figure S1. Biophysical properties of PBAE nanoparticles encapsulating varied DNA plasmids. Figure S2. Correlation between plasmid size and transfection efficacy of six HCC lines. Figure S3. Correlation between plasmid size and transfection efficacy of all cells. Figure S4. Correlation between plasmid size and GFP gMFI of six HCC lines. Figure S5. Correlation between plasmid size and GFP gMFI of all cells. (PDF) [file pone.0352468.s003.pdf]

Supporting Information

Engineering of Episomal Plasmid Structure to Enhance Non-viral Poly(beta-amino ester) Nanoparticle Gene Delivery to Liver and Brain Cancer Cells

Authors:
Joanna Yang[1,2], Jack Kollings[1,2], Ethan Idnani[1,2], David R. Wilson[1,2], Isabella G. Cozzone[1,2], Shanelle Mendes[1,2], Mahita Varanasi[1,2], Stephany Y. Tzeng[1,2,*], Jordan J. Green[1,2,3,4,5,6,7,8,*]

Affiliations:
1. Department of Biomedical Engineering, Johns Hopkins University School of Medicine, Baltimore, MD 21231, USA
2. Translational ImmunoEngineering Center, Translational Therapeutics and Regenerative Engineering Center, Johns Hopkins University School of Medicine, Baltimore, MD 21231, USA
3. Department of Chemical & Biomolecular Engineering, Johns Hopkins University, Baltimore, MD 21218, USA
4. Department of Oncology and the Sidney Kimmel Comprehensive Cancer Center, Johns Hopkins University School of Medicine, Baltimore, MD 21231, USA
5. Department of Ophthalmology, Johns Hopkins University School of Medicine, Baltimore, MD 21231, USA
6. Department of Neurosurgery, Johns Hopkins University School of Medicine, Baltimore, MD 21205, USA
7. Department of Materials Science & Engineering, Johns Hopkins University, Baltimore, MD 21218, USA
8. Institute for NanoBioTechnology and the Bloomberg~Kimmel Institute for Cancer Immunotherapy, Johns Hopkins University, Baltimore, MD 21231, USA
* To whom correspondence should be addressed: green@jhu.edu, stzeng1@jhmi.edu

Contents:

Table S1: Clonal gene sequences ordered from Twist Biosciences
Table S2: Detailed statistical analysis
Figure S1. Biophysical properties of PBAE nanoparticles encapsulating varied DNA plasmids
Figure S2. Correlation between plasmid size and transfection efficacy of six HCC lines
Figure S3. Correlation between plasmid size and transfection efficacy of all cells
Figure S4. Correlation between plasmid size and GFP gMFI of six HCC lines
Figure S5. Correlation between plasmid size and GFP gMFI of all cells

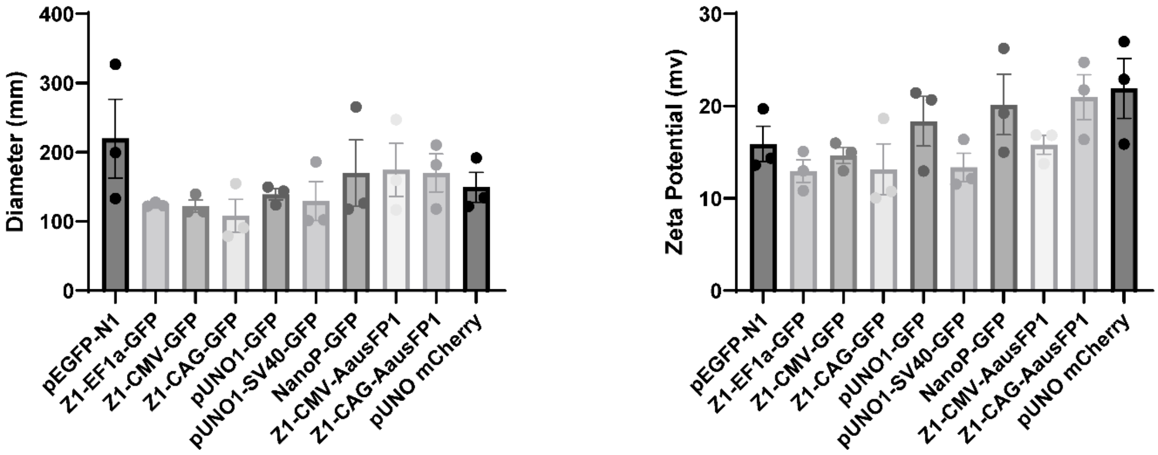

Figure S1. Biophysical properties of PBAE nanoparticles encapsulating varied DNA plasmids. Nanoparticle hydrodynamic diameters and zeta potentials were evaluated for each plasmid using the same formulation conditions and DNA mass. One-way ANOVA with Tukey's post tests were performed to evaluate statistical differences between plasmid types. No statistical differences were found between any of the groups for either hydrodynamic diameter or zeta potential ($P > 0.05$). Data bars shown represent mean ± SEM, n=3 independently fabricated nanoparticle replicates.

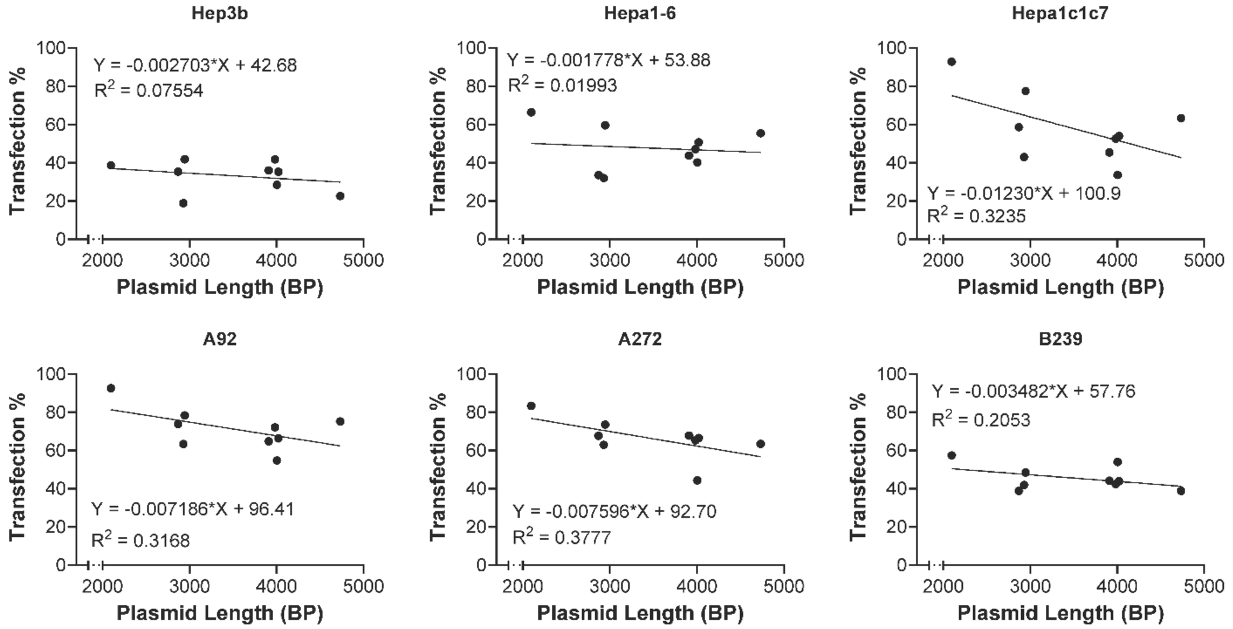

Figure S2. Correlation between plasmid size and transfection efficacy of six HCC lines.

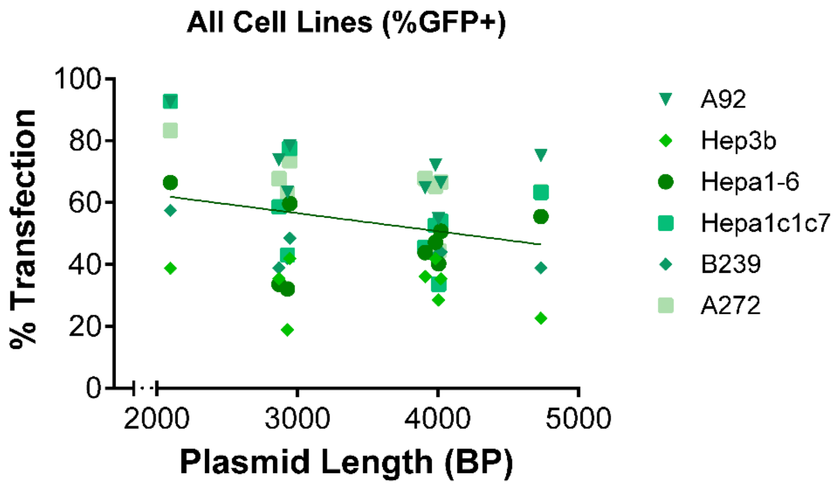

Y = -0.005841*X + 74.06 R^2 = 0.07244

Figure S3. Correlation between plasmid size and transfection efficacy of all cells.

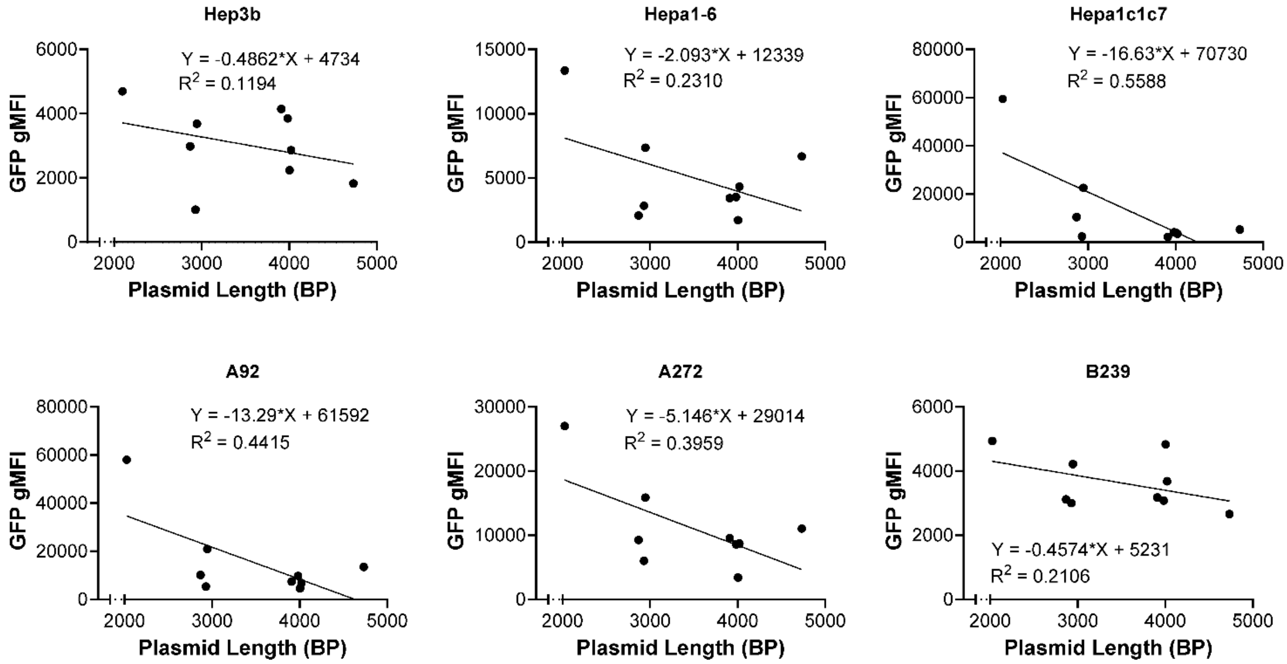

Figure S4. Correlation between plasmid size and GFP gMFI of six HCC lines.

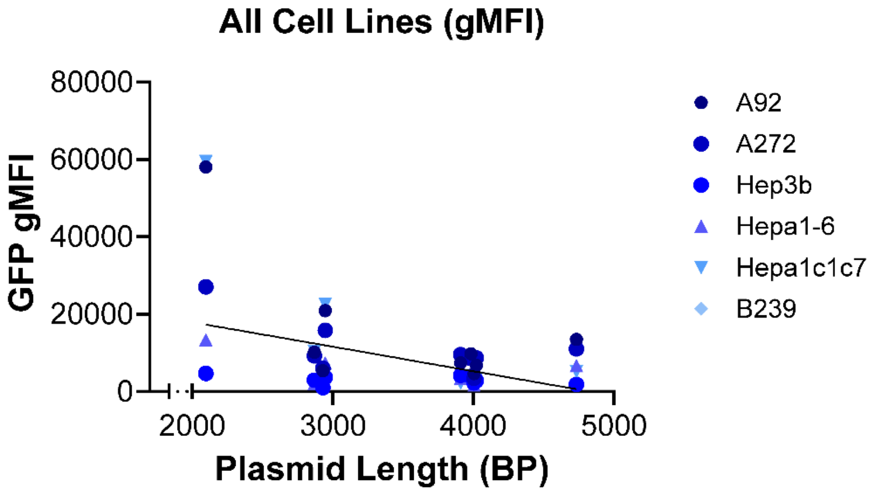

Y = -6.336*X + 30601	R^2 = 0.1938

Figure S5. Correlation between plasmid size and GFP gMFI of all cells.
